# Research impact: defining it, measuring it, maximising it, questioning it

**DOI:** 10.1186/1472-6963-14-S2-O30

**Published:** 2014-07-07

**Authors:** Trisha Greenhalgh

**Affiliations:** 1 Barts and The London School of Medicine and Dentistry, London, UK

## 

Both in the UK and more widely, the higher education sector is under pressure from the government to demonstrate that it makes a difference. That aside, few of us want to be the kind of academic that sits in an ivory tower thinking clever thoughts while Rome burns below. As Karl Marx said, many academics only interpret the world, but the true purpose of scholarship is to change it. The ‘impact agenda’ thus contains two goals that may occasionally be in tension with one another. The first is to demonstrate impact as defined by a relatively narrow set of government-driven criteria and metrics (most notably in the UK, the ‘Impact’ section of the Research Excellence Framework). There is much emphasis in policy circles, for example, on the need for medical schools to build links with industry with a view to generating ‘health and wealth’ (that is, improving survival or quality of life while also saving money and boosting business for our national industries). The second goal, more in line with the vision of engaged scholarship articulated by Marx, is to align the research agenda with such things as community-campus partnerships and a commitment to social justice. Whilst it is easy to be cynical about the colonisation of academia by commercial interests, many universities now boast fruitful industry collaborations that have not only supported the development of new drugs, devices and technologies (and, in a few cases, led to measurable economic benefit for the university) but which have also accelerated the uptake and use of innovations for societal benefit. Nevertheless, much of the impact from biomedical research to date has been through a wide range of *non-commercial* partnerships with clinical provider organizations, national policymakers, councils, schools, faith groups, third-sector organizations and citizens. In short, ‘impact’ is much more than commercial spin-offs.

As the first ‘Dean for Research Impact’ appointed by a UK higher education institution, Professor Greenhalgh will summarise prevailing national and international debates about what research impact is; how it should be measured; how to balance the potentially conflicting agendas of ‘economic’ and ‘societal’ impact; and how to build capacity at all levels for delivering on both these agendas.

